# Optimization of Spiral MRI Using a Perceptual Difference Model

**DOI:** 10.1155/IJBI/2006/35290

**Published:** 2006-10-09

**Authors:** Donglai Huo, Kyle A. Salem, Yuhao Jiang, David L. Wilson

**Affiliations:** ^1^ Department of Biomedical Engineering, Case Western Reserve University, Cleveland, OH 44106, USA; ^2^ Department of Radiology, University Hospitals of Cleveland, Case Western Reserve University, Cleveland, OH 44106-7055, USA

## Abstract

We systematically evaluated a variety of MR spiral imaging acquisition and reconstruction schemes using a computational perceptual difference model (PDM) that models the ability of humans to perceive a visual difference between a degraded “fast” MRI image with subsampling of *k*-space and a “gold standard” image mimicking full acquisition. Human subject experiments performed using a modified double-stimulus continuous-quality scale (DSCQS) correlated well with PDM, over a variety of images. In a smaller set of conditions, PDM scores agreed very well with human detectability measurements of image quality. Having validated the technique, PDM was used to systematically evaluate 2016 spiral image conditions (six interleave patterns, seven sampling densities, three density compensation schemes, four reconstruction methods, and four noise levels). Voronoi (VOR) with conventional regridding gave the best reconstructions. At a fixed sampling density, more interleaves gave better results. With noise present more interleaves and samples were desirable. With PDM, conditions were determined where equivalent image quality was obtained with 50% sampling in noise-free conditions. We conclude that PDM scoring provides an objective, useful tool for the assessment of fast MR image quality that can greatly aid the design of MR acquisition and signal processing strategies.


Hindawi Publishing Corporation
International Journal of Biomedical Imaging
Volume 2006, Article ID 35290, Pages 1–11
DOI 10.1155/IJBI/2006/35290

### Optimization of Spiral MRI Using a Perceptual Difference Model

**Donglai Huo,[1] Kyle A. Salem,[1] Yuhao Jiang,[1] and David L. Wilson[1,2]**

[1] *Department of Biomedical Engineering, Case Western Reserve University, Cleveland, OH 44106, USA*
[2] *Department of Radiology, University Hospitals of Cleveland, Case Western Reserve University, Cleveland, OH 44106-7055, USA*

Received 27 August 2005; Revised 26 June 2006; Accepted 6 July 2006

Recommended for Publication by Yibin Zheng

We systematically evaluated a variety of MR spiral imaging acquisition and reconstruction schemes using a computational perceptual difference model (PDM) that models the ability of humans to perceive a visual difference between a degraded "fast" MRI image with subsampling of $k$-space and a "gold standard" image mimicking full acquisition. Human subject experiments performed using a modified double-stimulus continuous-quality scale (DSCQS) correlated well with PDM, over a variety of images. In a smaller set of conditions, PDM scores agreed very well with human detectability measurements of image quality. Having validated the technique, PDM was used to systematically evaluate 2016 spiral image conditions (six interleave patterns, seven sampling densities, three density compensation schemes, four reconstruction methods, and four noise levels). Voronoi (VOR) with conventional regridding gave the best reconstructions. At a fixed sampling density, more interleaves gave better results. With noise present more interleaves and samples were desirable. With PDM, conditions were determined where equivalent image quality was obtained with 50% sampling in noise-free conditions. We conclude that PDM scoring provides an objective, useful tool for the assessment of fast MR image quality that can greatly aid the design of MR acquisition and signal processing strategies.

Copyright © 2006 Donglai Huo et al. This is an open access article distributed under the Creative Commons Attribution License, which permits unrestricted use, distribution, and reproduction in any medium, provided the original work is properly cited.

### 1. INTRODUCTION

There is significant effort to speed MR imaging with techniques such as keyhole imaging [1–3], wavelet imaging [4, 5], radial [6, 7] and spiral acquisitions [8–10], and parallel imaging [11, 12]. Spiral imaging is an effective and widely used fast MRI technique with a number of advantages. It traverses the $k$-space very efficiently; it has superior flow and motion characteristics due to the fact that the trajectory starts from the $k$-space center, thus providing gradient moment compensation to all orders. It has been widely used in flow imaging [9], functional MRI [10, 13], and cardiac imaging [8, 14]. However, one disadvantage of spiral MRI is the need for a nontrivial reconstruction method, since data are not acquired on a rectilinear grid.

Several methods have been proposed to reconstruct spiral images. The first and most commonly cited method described by Meyer et al. [8] and Jackson et al. [15] is often referred to as conventional regridding. This method interpolates the nonuniform data to a rectilinear grid before Fourier reconstruction. Another method is matrix resampling (MXR), proposed by Oesterle et al. [16]. This method places nonuniform data onto an over sampled uniform grid of varying size by nearest neighbor interpolation before Fourier inversion. There are other methods, such as the direct summation method, which is not practical due to the high computational demand; the block uniform resampling method [17], which has the difficulties with low SNR data. We do not compare these latter methods in this paper.

Most reconstruction methods require the use of a density compensation function (DCF) to account for the nonuniform density of spiral sampling. Researchers have proposed a number of different DCF implementations. In this paper, we will focus on three methods, area density function (ADF) [8, 15], Voronoi diagram [18], and simplified Jacobian determinant (SJD) method [19]. Detailed descriptions of these algorithms have appeared in the literature, including our previous paper [20].

There are also acquisition parameters to be chosen for spiral acquisitions. One must decide between single-shot or interleaved spirals. The single-shot method has limited signal-to-noise ratio (SNR) and resolution due to T2* decay; while the interleaved spiral sequences increase the total imaging time. One also must choose the number of samples

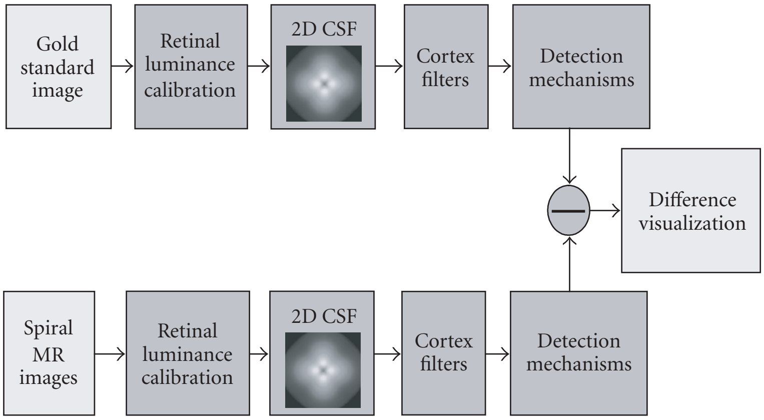

Figure 1: Perceptual difference model (PDM). The output is a map showing the likelihood of a perceptual difference between the two input images. The gold standard image is Shepp and Logan phantom reconstructed with ideal Cartesian $k$-space data. Subsequent test images are reconstructed from different spiral acquisition parameters and reconstruction methods at different noise levels. CSF refers to the contrast sensitivity function, which describes how sensitive human eyes are to various frequencies of visual stimuli.

to acquire. Investigators have used a full range from 200% [21] to $\approx$ 60% [16] of the number of samples on a rectilinear grid with the same radius. With different pulse sequences, the SNR of acquired data can change significantly. Hence, one must determine the sensitivity of acquisition and reconstruction parameters to noise.

Combinations of acquisition and reconstruction parameters can easily generate thousands of images, creating a very difficult task for human review. For objective assessment of image quality, we use a computer perceptual difference model (PDM), which predicts the degree to which a human observer can detect differences between two images. The block diagram is shown in Figure 1. Researchers have used different forms of spatial and spatiotemporal visual models similar to PDM to assess the image quality of digitally coded, compressed pictures and image sequences [22], evaluate image display quality [23], detect tumors [24], and micro-calcifications in mammography [25], evaluate compression algorithms [26], and develop image processing algorithms, imaging system hardware, and imaging media [27]. Recently, we developed the PDM, validated it against human scoring, and used it to evaluate keyhole imaging parameters and assess the quality of fat suppression [28–31].

In this paper, we use PDM to study MR spiral imaging using an analytical phantom that allows one to obtain exact $k$-space samples along a spiral. We first describe the PDM method and experiments to compare PDM to human observer assessment in a modified double-stimulus continuous-quality scale (DSCQS) experiment, which measures the quality of an image relative to a reference as recommended by the International Telecommunication Union. In addition, we compare PDM results to human detection measures from an adaptive forced choice study (AFC). This is a standard approach for objective measurement of image quality that has been extensively applied in nuclear and X-ray imaging by many, including our laboratory [32, 33]. Following these validation experiments, we performed a systematic evaluation of 2016 spiral imaging conditions using multiple MR data sets and independent variables consisting of acquisition parameters, reconstruction methods, and noise. Finally, data are analyzed to provide recommendations for spiral imaging.

### 2. METHODS

### 2.1. Spiral image simulation

Spiral MR images were simulated using a version of an analytical Shepp and Logan phantom as shown in Figure 2(b) and described in [34]. The analytical Fourier transform of the phantom is known, making it possible to obtain exact sample values at any location in the Fourier domain. Ellipse intensities have been chosen and T2* (40 ms, 25 ms, and 10 ms for different ellipses) decay has been imposed on 6 of the ellipses to make the simulation more realistic. Data values were sampled from different spiral trajectories, all of which were Archimedean [35] spirals in $k$-space, which in polar coordinates $(r, \theta)$ can be described by the equation $r = a + b\theta$.

To design spirals, the numerical technique proposed in [36] was employed that minimizes the time required to traverse the spiral for given hardware specifications. We simulated a Siemens Magnetom Sonata 1.5 T MR imager with maximum gradient amplitude of 40 mT/m and slew-rate of 200 T/m/s. All trajectories were designed to have the same total sampling time (90 ms) and to reach the same maximum $k$-space radius (4445 $m^{-1}$). This resulted in 64 turns for the single-shot 90 ms spiral. In our experiments, the 42 trajectories were created from six different interleave patterns (1, 5, 9, 13, 17, and 21) and seven different sampling densities with total number of points ranging from 6552 to 16385 (approximately 40% to 100% of the number of samples in the $128 \times 128$ Cartesian phantom). The ratio of the size of the circular sampling region for the spirals to the square Cartesian grid is $\pi/4$, or 0.785.

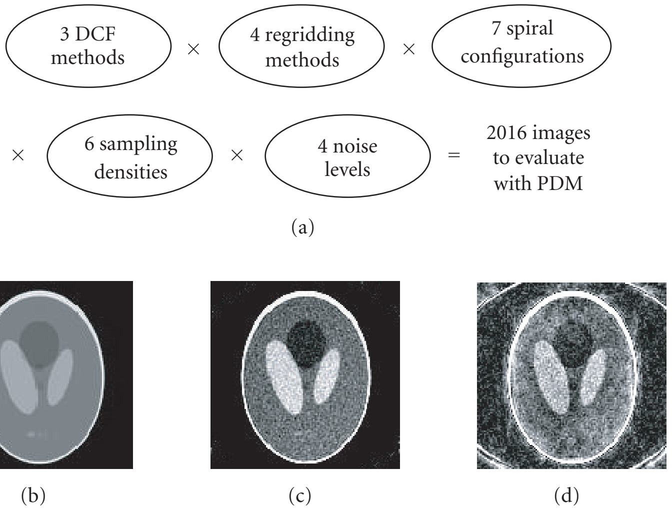

FIGURE 2: Many images of varying quality were systematically evaluated with PDM. We evaluated the 2016 conditions identified in (a). The original, or reference, image is shown in (b), with a PDM score of 0. The image in (c) was acquired with 21 interleaves, 100% sampling density, noise level = 3, and reconstructed with Voronoi and conventional regridding. This is an example of good image quality for a noisy image, giving a PDM score of 14.34. With 5 interleaves, 80% sampling density, noise level = 3, a reconstruction with ADF and MXR 2X gives particularly poor image quality (d) with a PDM score of 21.47.

### 2.2. Adding noise

Simulating a single transmission/receiver coil with perfectly uniform characteristics, noise was added to the noise-free, Fourier domain data. As described in [37], we added Gaussian-distributed, zero-mean white noise to both the real and imaginary channels in the $k$-space. Noise with different standard deviations (3, 5, and 10) was added to the spiral $k$-space data. These noise levels when added to Cartesian $k$-space data give image-space SNRs of 17, 10 and 5, respectively.

### 2.3. Image reconstruction

As shown in Figure 2(a), from each of the 42 spiral trajectories (7 spiral configurations and 6 sampling densities), 12 simulated images were reconstructed from the sampled data. The 12 reconstructions come from three DCF options (ADF, Voronoi, or SJD) and four reconstruction methods (MXR 2X, MXR 4X, MXR 8X, and conventional regridding). One hundred noise realizations were created for each noisy image, making a total of $7*6*4*3*100*3 = 151\,200$ images. PDM scores for similar noise were averaged.

The ADF was calculated with a convolution kernel window width of 3.0, and the Kaiser-Bessel free parameter value was set to minimize the relative aliased energy according to the guidelines in [15]. The Voronoi and SJD were calculated for each individual trajectory using the $k$-space and, for the SJD, gradient values. The three DCF processed data sets for each trajectory were then used to reconstruct four different images. Conventional regridding was used with a Kaiser-Bessel window width of 3.0, and the MXR procedure was employed using three different over-sampling factors, 2X, 4X, and 8X. All reconstructions were performed on a 3 GHz Pentium 4 PC (Dell Computer, Austin, Tex) using Matlab (The MathWorks, Natick, Mass) code written in our laboratory.

### 2.4. Image evaluation with PDM

Images were compared using a perceptual difference model (PDM) designed in our laboratory and described in detail and validated for the evaluation of fast MRI applications elsewhere [28, 30, 31, 38]. It contains components that model the nonlinearity in the sensitivity of the retina [39, 40], the contrast sensitivity function [39], and the channels of spatial frequency found in the visual cortex [41], as well as other features including a measure of contrast [27], and visual detection threshold [42]. The structure of PDM is shown in Figure 1 and detailed explanation has been published in [28]. Inputs to the PDM were an ideal reference image obtained from the Cartesian sampled original phantom and one of the 2016 degraded spiral images. Images were windowed to maximize the overall image contrast, and this same windowing was maintained during evaluation by human observers described later. The output of the PDM was a spatial map representing the magnitude of differences that a human observer would perceive between the two images. This map can be summed over a region of interest (ROI), defined manually to include relevant anatomy, to give a scalar PDM error. In this paper, we used an ROI consisting of a manually defined ellipse encompassing the large bright rim (inner) of the phantom and including all other ellipses.

### 2.5. Comparison of PDM to human evaluation

Image quality scores of selected images were determined using a modified double-stimulus continuous quality-scale (DSCQS) test [43], which is very similar to that previously

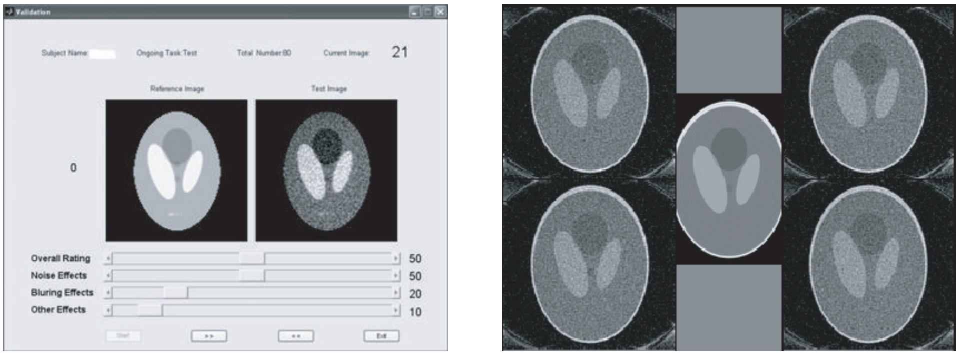

(a) (b)

Figure 3: Screen shots showing methods for human subject evaluation of image quality. In the modified DSCQS experiment (a), subjects scored a test image relative to the reference. Note that only the region of interest over the "head" is shown and that other regions are blocked. In the adaptive 4-AFC experiment (b), the subject must choose which of the four panels contains the lesion and mark it with a cursor. (The answer is the lower left-hand corner.) The center image is a reference showing the location of the lesion.

reported by Salem et al. [28] and by Martens and Meesters on a similar model used for other purposes [44]. To test the full range of image quality in our simulated images, we selected 40 test images with PDM scores uniformly spread from best to worst. The 40 images were presented to three subjects, one of the authors and two MRI experts. The image was displayed following gray scale windowing as reported previously. The region outside of the region of interest was set to zero value (black). The evaluation experiment was carried out on a Matlab GUI program (Figure 3(a)), and all the results were automatically recorded. Each presentation consisted of a two-panel display, with the high-quality reference image and a randomly selected test image on the left and right, respectively. Observers were instructed to score the quality of the test image on a scale of 100 to 0, with 0 being the best quality and 100 being the worst quality, by sliding a slider with mouse or keyboard. We made observers aware that we considered the reference image to be "best" and they should consider it to have a score of 0. Three ratings were obtained for each image pair. That is, we asked observers to rate the test image on: (1) overall image quality, (2) "noise effects," and (3) aliasing and other reconstruction errors. In a training session, the two observers naïve to hypotheses were shown a wide range of images and instructed as to what we considered "noise" (high frequency, relatively uncorrelated noise) and "aliasing and other reconstruction errors." Following this discussion on at least 10 images, observers performed a training session on at least 30 images spanning a wide range of image quality, so as to help calibrate them for the experiment. During this time, subjects were free to ask questions of the first author. To account for intraobserver differences, each of the 40 test images was displayed and evaluated twice. The experiment was carried out in a darkened room and normally took 1 hour. A perceptually linearized, high quality gray scale monitor was used. There was no time limitation in the experiment, and subjects were allowed to revise their results, including back-tracking, at any time.

Data were processed before comparisons to PDM. First, two scores given for the same test image from the same subject were averaged to reduce the intra-observer variability. To compensate for scale boundary effects, a non-linear scale transformation was used, as recommended by the International Telecommunication Union in their report on methods for assessing television images [43]. The transformation is represented by the following equations, in which $\overline{u}$ and $u_{\text{corr}}$ represent the scores before and after transformation, respectively; $u_{\min}$ and $u_{\max}$ are the boundaries of the after-transformation scores; $u_{\text{mid}}$ is the middle of the after-transformation score, and $u_{0\min}$, $u_{0\max}$ are the lower and upper boundaries of the before-transformation scores:

$$u_{\text{corr}} = C(\overline{u} - u_{\text{mid}}) + u_{\text{mid}},$$

$$C = \frac{\overline{u} - u_{0\min}}{u_{0\max} - u_{0\min}} \frac{u_{\max} - u_{\text{mid}}}{u_{0\max} - u_{\text{mid}}} + \frac{u_{0\max} - \overline{u}}{u_{0\max} - u_{0\min}} \frac{u_{\min} - u_{\text{mid}}}{u_{0\min} - u_{\text{mid}}}. \tag{1}$$

### 2.6. Detection 4-AFC experiment

Human observer detection experiments were also performed to assess image quality. The ability to detect lesions, oftentimes simulated lesion, has long been seen as a desirable task-oriented method to evaluate the quality of noise-dominant images, such as X-ray images. Two main experimental methods to measure detectability have been used: the receiver operating characteristic (ROC) test and the alternative forced choice (AFC) test [32, 45]. We used an adaptive 4-AFC paradigm, which has advantages [46, 47] for our well-controlled phantom experiment. We performed experiments using the three spiral schemes and one Cartesian scheme listed below. The spiral schemes span different DCF methods and sampling densities.

(1) DCF = ADF, regridding = conventional regridding, number of interleaves = 5, sampling = 100%, noise level = 3.
(2) DCF = VOR, regridding = conventional regridding, number of interleaves = 5, sampling = 90%, noise level = 3.

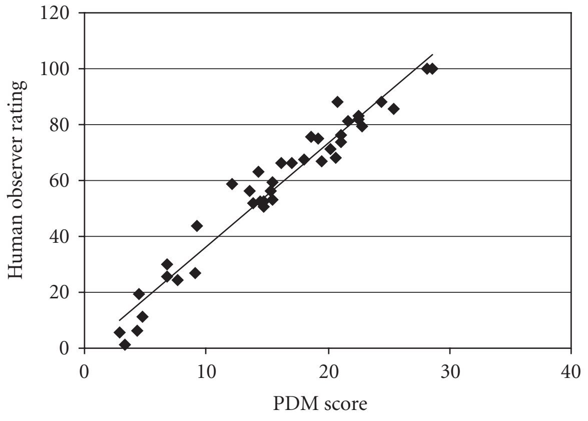

Figure 4: For the human subject evaluation experiment shown in Figure 3(a), normalized human subject ratings are plotted as a function of PDM score. Data were fit with $y = mx + b$, where $m = 3.69 + -0.27$ and $b = -0.48 + -4.78$, giving $R = 0.97$.

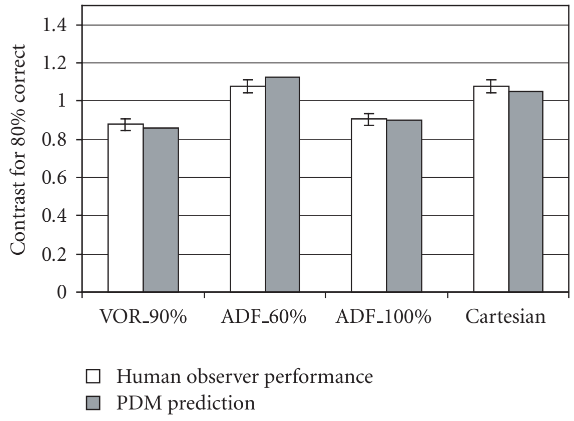

Figure 5: Comparison between human detection in the 4-AFC experiment and scaled PDM scores. PDM scores correlate surprisingly well with this task-oriented measure of image quality. We chose four interesting conditions that include the effect of DCFs and sampling densities. Other parameters are conventional regridding, 5 interleaves, and noise = 3. The Cartesian case is added for comparison. Detailed conditions for experiments are described in Section 2.

(3) DCF = VOR, regridding = conventional regridding, number of interleaves = 5, sampling = 60%, noise level = 3.
(4) Cartesian acquisition, sampling = 100%, noise level = 3.

In the 4-AFC experiments, we presented four noisy images obtained under the same imaging conditions on the monitor (Figure 3(b)). The target, or signal to be detected, was a dark ellipse always located at a fixed position near the middle of the image. The target was present in only one of the four panels, and the panel varied randomly from one trial to the next. A noise-free, signal-present image was displayed in the center as a reference. The subject correctly or incorrectly chose the panel containing the ellipse. As described in detail elsewhere [32], target contrast was adjusted each time adaptively using a maximum-likelihood technique based on the previous responses, so that there was an 80% probability of correct detection. For a 4-AFC experiment, this corresponds to $d'_{80\%} = 1.893$ [48]. Performance level was therefore fixed, and the output was the final contrast in terms of a change in gray level in our 8-bit images. Standard errors were estimated using a method that accounted for adaptation [32]. Subjects were trained for 100 trials before obtaining the data. Experiments were performed in a darkened room, included 300 trials, and took about 1 hour to complete.

### 3. RESULTS

We compared PDM scores to processed human subject ratings from the DSCQS test (Figure 4). Human observer scoring of image quality was highly correlated with PDM scores ($R = 0.97, p < 0.001$). For comparison, an alternative metric, the mean square error (MSE) between the test and reference images, gave a much poorer correlation ($R = 0.86, p < 0.001$).

For selected interesting conditions, we performed human detection experiments using a 4-AFC experiment described in Section 2. We chose three interesting spiral conditions that include the effect of DCFs and sampling densities. Other parameters are conventional regridding, 5 interleaves, and noise = 3. The Cartesian case was added for comparison. Contrasts for 80% probability correct are plotted in Figure 5. VOR_90% gives the lowest contrast, and the best image quality. ADF_100% gave very similar results. There is remarkable agreement with PDM scores, which were computed as described in Section 2 and linearly scaled to fit with the contrast values.

As described in Section 2, we investigated a variety of acquisition and reconstruction parameters, giving 2016 conditions. PDM and selected images were examined in detail. Because of the size of the parameter space, only selected results will be shown. For the noise conditions, there were 100 noise realizations for each image, and the PDM scores were the average of these 100 realizations. Standard deviation is less than 1% and not shown in the figures.

Reconstruction methods are first analyzed (Figure 6). PDM scores are plotted as a function of reconstruction and acquisition parameters and noise. With regards to density compensation methods, VOR is almost the same as ADF, and both give lower PDM scores than SJD. As for regridding methods, conventional is slightly better than MXR 8X, and both are advantageous as compared to MXR 2X and MXR 4X.

To more comprehensively compare the effect of a variable, we collapsed PDM scores by averaging them over all other parameters. For example, in Figure 7(a), we plot PDM scores averaged in this way as a function of density compensation methods. We again see that VOR and ADF work better than SJD. To further compare VOR and ADF, we determined the numbers of times that each method “wins” by having a smaller PDM score (Figure 7(b)). Results are shown for these two DCFs as a function of noise collapsed over all

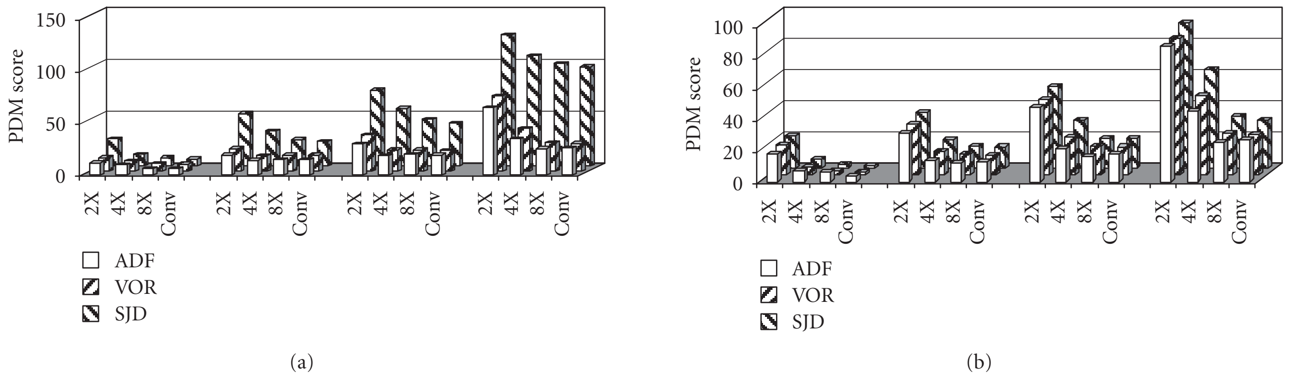

Figure 6: Reconstruction methods with different noise levels and sampling densities. PDM scores are plotted as a function of different DCFs and regridding methods, at 4 noise levels. In (a), images were acquired with a single-shot and sampling density = 0.8. In (b), images were acquired with interleaves = 17 and sampling density = 0.9.

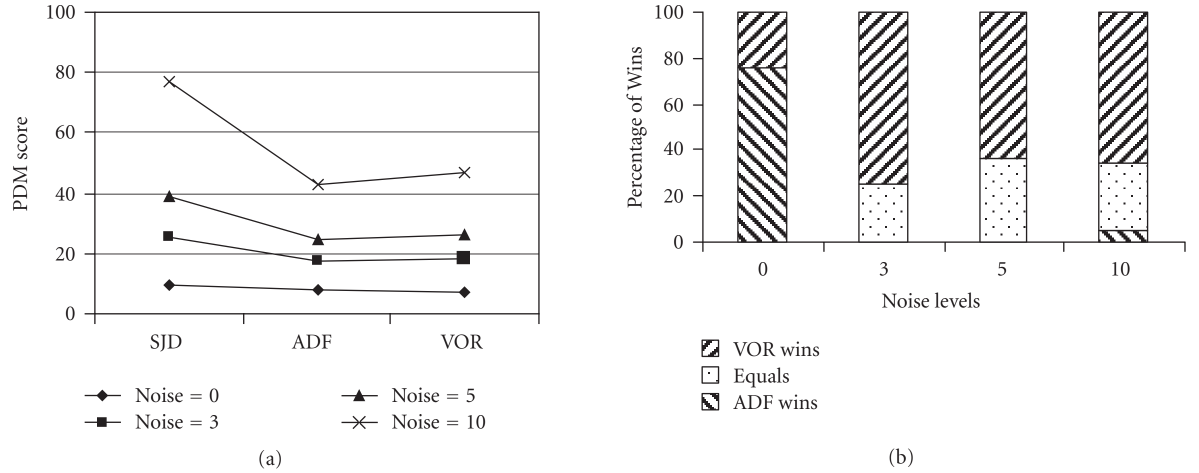

Figure 7: The comparison of density compensation functions. The average performance of three density compensation functions under different noise conditions is shown in the logarithm chart in (a). ADF and VOR show obvious advantages over SJD. ADF and VOR are compared case by case in same conditions, and the statistical results are shown in (b). For noise conditions, we have tested 100 realizations for each image, and the PDM scores are statistically analyzed. In (b), the "equals" are observed when a $t$ test shows no significant difference ($p > 0.05$). This does not apply to the noise-free condition.

other 168 conditions (4 regridding methods, 42 spiral trajectories). ADF beats VOR in noise-free conditions, but VOR performs better in noise. Considering that VOR is computationally more efficient than ADF and that some noise is always present, it is reasonable to use VOR.

Similarly, effects of regridding methods are shown in Figure 8(a), where PDM scores averaged as described above are plotted. MXR 8X and conventional regridding work better than the MXR 4X and MXR 2X. Further comparison of conventional regridding and MXR 8X is shown in Figure 8(b) as a function of noise, where results are collapsed over the 126 other conditions (3 DCFs and 42 spiral trajectories). MXR 8X beats conventional regridding in the noise-free condition, but conventional regridding performs a little better with noise present. Considering that some noise is always present and the popularity of conventional regridding, we recommend it.

We now investigate the selection of acquisition parameters using VOR and conventional regridding for reconstruction. In Figures 9 and 10, we plot PDM score as a function of the number of interleaves and sampling densities, with no noise and noise = 3, respectively. PDM scores under noise-free conditions (Figure 9(a)) are very different than those with added noise (Figure 10(a)). Absolute PDM scores are very much degraded with added noise. For noise-free conditions, the single shot (number of interleaves = 1) acquisition gives a much worse result than cases with more interleaves. With added noise, this effect is not so large. Both with and without noise, more interleaves always lead to better image quality.

Image quality also depends on sampling density. From Figure 9(a), in noise-free conditions, the image quality does not decrease too much with the decrease sampling density, until the sampling density reaches 50%. We can see the effects

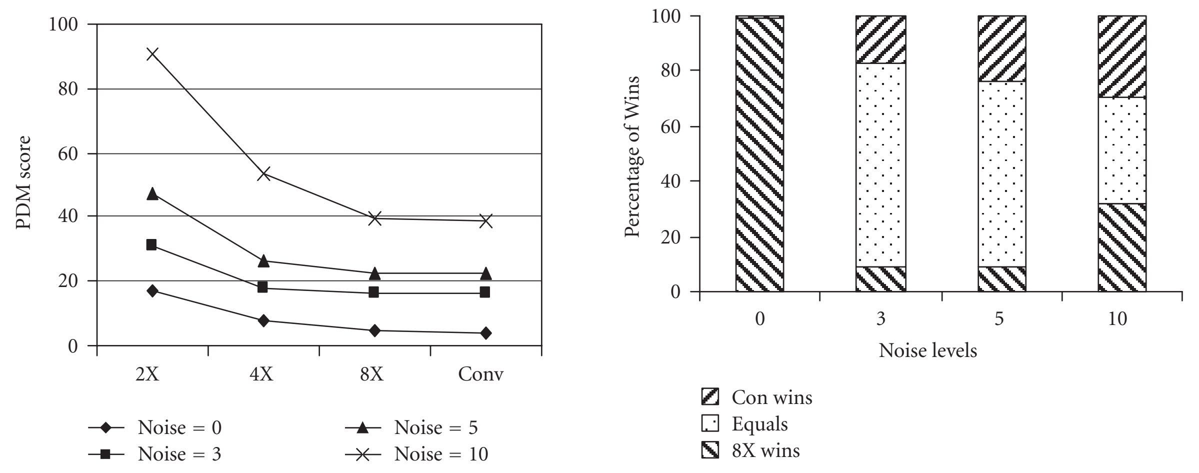

(a) (b)

Figure 8: The comparison of regridding methods. The average performance of four regridding methods under different noise conditions is shown in (a). Conventional regridding and MXR 8X show obvious advantages over MXR 2X and MXR 4X. Conventional regridding and MXR 8X are compared case by case in same conditions and the statistical results are shown in (b). For noise conditions, we have tested 100 realizations for each image, and the PDM scores are statistically analyzed. In (b), the "equals" are only applied in the meaning of statistic ($t$ test with 0.95 confidence interval) and do not apply to noise-free condition.

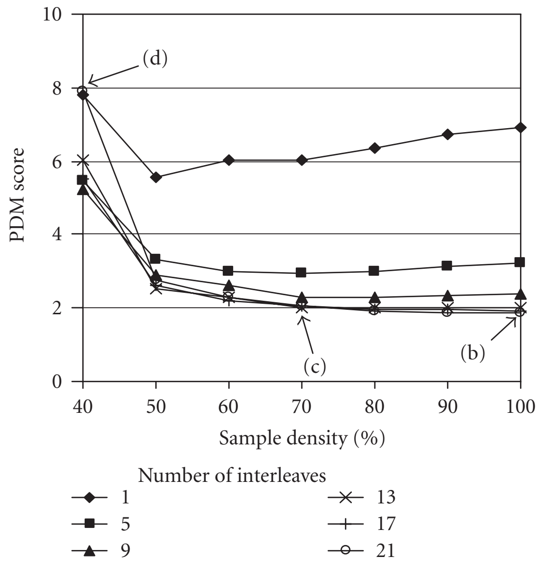

(a)

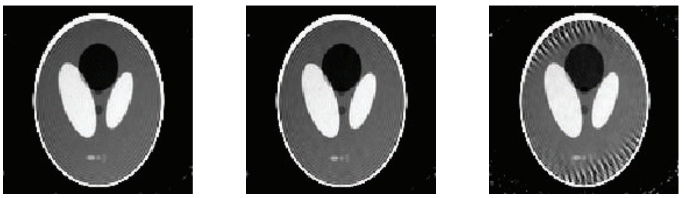

(b) (c) (d)

Figure 9: Effect of number of interleaves and sampling densities. In (a), PDM is plotted as a function of the sampling densities from 40% to 100% and 6 different numbers of interleaves. Other conditions (Voronoi method, conventional regridding, noise-free) were held constant. Images in (b), (c), and (d) correspond to 100% sample, 70% sample, and 40% sample and 21 interleaves, respectively, as indicated.

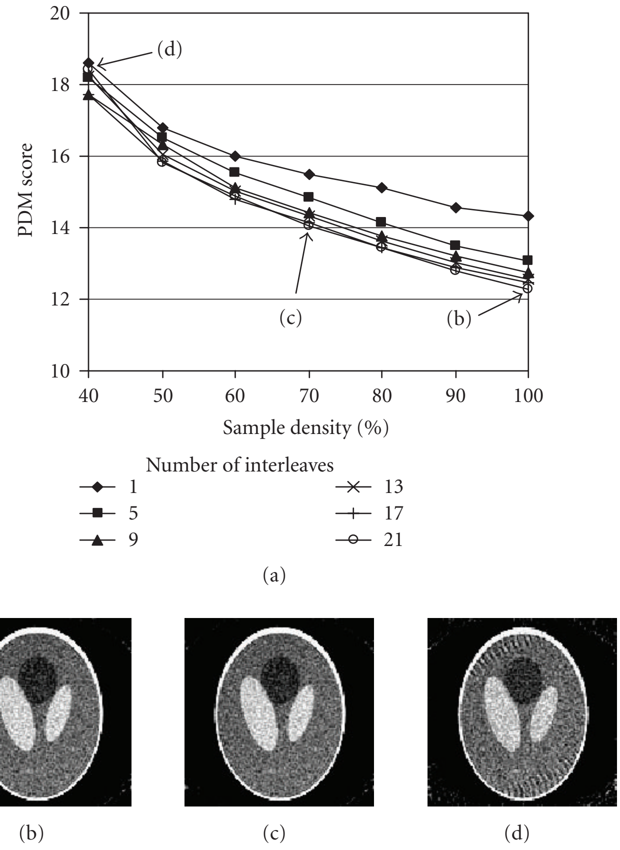

Figure 10: Effect of number of interleaves and sampling densities. In (a), PDM is plotted as a function of the sampling densities from 40% to 100% and 6 different numbers of interleaves. Other conditions (Voronoi method, conventional regridding, noise = 3) were held constant. Images in (b), (c), and (d) correspond to 100% sample, 70% sample, and 40% sample and 21 interleaves, respectively, as indicated.

from the comparison of Figures 9(b) and 9(c). No significant difference on image quality can be identified between 100% sampling and 70% sampling. When the sampling level reaches 40% as shown in Figure 9(d), the reconstruction image contains obvious structure artifact. Comparably, under noise conditions in Figure 10(a), we can see that the image quality keeps falling with the decrease of sampling density. Compare with Figures 9(c) and 10(b), which are 100% sampling and 70% sampling in noise conditions, it is obvious to see that the 70% sampling image is much noisier.

### 4. DISCUSSION

The evaluation of image quality in MR spiral imaging presents a unique challenge for existing methods since images are degraded by several factors and since so many different imaging techniques are possible. Fast acquisitions will induce noise and artifacts such as blur incurred by the off-resonance effects and ringing from the complex reconstruction process. As a result, traditional assessments such as MSE or SNR do not correctly predict the image quality, as shown in our previous experiments [28]. Detection studies are one alternative for evaluation of image quality, especially in those instances where "detection" is the goal. The time and effort for human detection studies over the thousands of possibilities for fast MR imaging make it unrealistic for image quality evaluation. For example, the 4-AFC study in this report only evaluated four imaging schemes, yet it took many person hours to complete. On the contrary, a PDM evaluation took less than 20 seconds and gave very similar results. Moreover, some reasons for doing MR imaging, such as guidance for intervention, measurement of parameters such as tumor volume, assessment of therapy, and so forth, do not all map well to the detection paradigm.

In this report, we extend the application of PDM image quality evaluation to spiral MR imaging. PDM makes it possible to rapidly evaluate the 1000's of possibilities with regards to image acquisition and reconstruction parameters. Important, sometimes surprising, results are obtained as outlined later. The good correlation between PDM and human subject scoring (Figure 4) and between PDM and human subject detection (Figure 5) is very encouraging.

The PDM scores were calculated based on the average degradation over a region of interest. Therefore, the PDM scores do not change much with different noise realizations. In this paper, although 100 measurements were simulated for each spiral image, analysis of the standard deviation shows that a single measurement will give accuracy within one percent.

We can make some recommendations for reconstruction based on our comprehensive simulations. We recommend the Voronoi method and conventional regridding. Even in those few cases that this combination did not give the best result, it always gave near-best results. Moreover, results were most often superior with added noise. Another option is the area density function as the DCF and MXR 8X as the regridding method, a combination which works almost as well as the recommended method. Based on our results, we do not recommend SJD as a density compensation function or MXR 2X, 4X as regridding methods.

We can also recommend acquisition parameters. A higher number of interleaves is always desirable, both with and without noise, at a given sampling density. There are at least two potential reasons. First, more interleaves decreases or avoids the effects of T2* blur. Second, with noise, a higher number of interleaves results in a higher sampling density in low-frequency *k*-space, which should help reduce low-frequency noise and its effect on image quality. It could also help to reduce off-resonance effects, which are not included in our simulation but are another concern in spiral imaging. From our experiments, we see that sampling densities as low as 50% can be used under noise-free conditions without significantly affecting image quality. With significant noise, more samples and more interleaves are always preferred (Figure 10). As a comparison, Figures 2(c) and 2(d) show how different parameters affect image quality.

It is uncommon to apply detection studies to MR images. Forced choice experiments have been commonly applied to studies of X-ray image quality, where quantum noise and background structures limit detection. In some instances, noise in MR is not as dominant as in X-ray imaging. However, reconstruction artifacts and clinical structures are always present. For those instances where detection of lesions is important, it seems quite appropriate to use detection studies to evaluate MR image quality.

No prior reports were found comparing PDM scores on MR images to human detection results. Task-orientated measures of image quality are desirable because they reduce the effect of user preference. Detection studies provide the most commonly used possibility. The remarkable agreement between PDM and detection is very encouraging. In this comparison, one should remember that detection studies are very time consuming, while PDM can be applied to thousands of images in a relatively short time.

It is interesting to compare spiral and conventional Cartesian imaging. In the detection study of the AFC experiment, we compared three spiral schemes and one Cartesian scheme, all at a moderate noise level of 3. Results are surprising as shown in Figure 5. The conventional Cartesian acquisition was inferior to spiral acquisitions even when fewer samples were obtained with the spiral. We believe this happens because the spiral acquisition leads to a higher sampling density in the center of *k*-space. This over-sampling suppresses the low-frequency noise which degrades image quality. Spiral imaging is most often touted because of its relative insensitivity to motion and flow. Our results suggest an advantage even with still data sets.

There are alternatives for using the PDM model. One potentially attractive idea is to use a "PDM score" emphasizing different spatial frequencies. In experiments, we collected human scores for noise, aliasing, and other effects. We found a good correlation between the high frequency output of the PDM and human scoring of noise (not shown). Similarly, the low-frequency output of the PDM is correlated with the combination of aliasing and other effects. Although further investigation is needed, PDM provides a possible way to separate such effects on image quality. Johnson et al. described similar observations with regards to the evaluation of parallel imaging techniques [49].

Although we offer recommendations, one must carefully apply results from a PDM phantom study directly to clinical use. Results will likely depend on the anatomy being imaged, which affects spatial frequency content. In the presence of moving anatomical structures, fast imaging can reduce motion artifacts; our comparisons do not take this into account. Nevertheless, the power of PDM lies in its ability to systematically rank many different images quickly and accurately. We believe that PDM can show the MR sequence designer the most appropriate options for further consideration.

### ACKNOWLEDGMENTS

This work was supported under NIH Grant R01 EB004070 and the Research Facilities Improvement Program Grant NIH C06RR12463-01. The authors thank Moriguchi Hisamoto in University Hospital of Cleveland for the discussion of spiral imaging, Meredith Heinzel for helping the editing, and the subjects for participating in the experiments.

### REFERENCES

[1] J. E. Bishop, G. E. Santyr, F. Kelcz, and D. B. Plewes, "Limitations of the keyhole technique for quantitative dynamic contrast-enhanced breast MRI," *Journal of Magnetic Resonance Imaging*, vol. 7, no. 4, pp. 716–723, 1997.

[2] J. L. Duerk, J. S. Lewin, and D. H. Wu, "Application of keyhole imaging to interventional MRI: a simulation study to predict sequence requirements," *Journal of Magnetic Resonance Imaging*, vol. 6, no. 6, pp. 918–924, 1996.

[3] J. J. van Vaals, M. E. Brummer, W. T. Dixon, et al., "Keyhole method for accelerating imaging of contrast agent uptake," *Journal of Magnetic Resonance Imaging*, vol. 3, no. 4, pp. 671–675, 1993.

[4] L. P. Panych, P. D. Jakab, and F. A. Jolesz, "Implementation of wavelet-encoded MR imaging," *Journal of Magnetic Resonance Imaging*, vol. 3, no. 4, pp. 649–655, 1993.

[5] J. B. Weaver, Y. Xu, D. M. Healy, and J. R. Driscoll, "Wavelet-encoded MR imaging," *Magnetic Resonance in Medicine*, vol. 24, no. 2, pp. 275–287, 1992.

[6] A. Shankaranarayanan, O. P. Simonetti, G. Laub, J. S. Lewin, and J. L. Duerk, "Segmented *k*-space and real-time cardiac cine MR imaging with radial trajectories," *Radiology*, vol. 221, no. 3, pp. 827–836, 2001.

[7] A. Shankaranarayanan, M. Wendt, A. J. Aschoff, J. S. Lewin, and J. L. Duerk, "Radial keyhole sequences for low field projection reconstruction interventional MRI," *Journal of Magnetic Resonance Imaging*, vol. 13, no. 1, pp. 142–151, 2001.

[8] C. H. Meyer, B. S. Hu, D. G. Nishimura, and A. Macovski, "Fast spiral coronary artery imaging," *Magnetic Resonance in Medicine*, vol. 28, no. 2, pp. 202–213, 1992.

[9] P. D. Gatehouse, D. N. Firmin, S. Collins, and D. B. Longmore, "Real time blood flow imaging by spiral scan phase velocity mapping," *Magnetic Resonance in Medicine*, vol. 31, no. 5, pp. 504–512, 1994.

[10] G. H. Glover and S. Lai, "Self-navigated spiral fMRI: interleaved versus single-shot," *Magnetic Resonance in Medicine*, vol. 39, no. 3, pp. 361–368, 1998.

[11] K. P. Pruessmann, M. Weiger, M. B. Scheidegger, and P. Boesiger, "SENSE: sensitivity encoding for fast MRI," *Magnetic Resonance in Medicine*, vol. 42, no. 5, pp. 952–962, 1999.

[12] D. K. Sodickson and W. J. Manning, "Simultaneous acquisition of spatial harmonics (SMASH): fast imaging with radiofrequency coil arrays," *Magnetic Resonance in Medicine*, vol. 38, no. 4, pp. 591–603, 1997.

[13] G. H. Glover and A. T. Lee, "Motion artifacts in fMRI: comparison of 2DFT with PR and spiral scan methods," *Magnetic Resonance in Medicine*, vol. 33, no. 5, pp. 624–635, 1995.

[14] P. Börnert, B. Aldefeld, and K. Nehrke, "Improved 3D spiral imaging for coronary MR angiography," *Magnetic Resonance in Medicine*, vol. 45, no. 1, pp. 172–175, 2001.

[15] J. I. Jackson, C. H. Meyer, D. G. Nishimura, and A. Macovski, "Selection of a convolution function for Fourier inversion using gridding," *IEEE Transactions on Medical Imaging*, vol. 10, no. 3, pp. 473–478, 1991.

[16] C. Oesterle, M. Markl, R. Strecker, F. M. Kraemer, and J. Hennig, "Spiral reconstruction by regridding to a large rectilinear matrix: a practical solution for routine systems," *Journal of Magnetic Resonance Imaging*, vol. 10, no. 1, pp. 84–92, 1999.

[17] D. Rosenfeld, "An optimal and efficient new gridding algorithm using singular value decomposition," *Magnetic Resonance in Medicine*, vol. 40, no. 1, pp. 14–23, 1998.

[18] V. Rasche, R. Proksa, R. Sinkus, P. Boernert, and H. Eggers, "Resampling of data between arbitrary grids using convolution interpolation," *IEEE Transactions on Medical Imaging*, vol. 18, no. 5, pp. 385–392, 1999.

[19] R. D. Hoge, R. K. S. Kwan, and G. B. Pike, "Density compensation functions for spiral MRI," *Magnetic Resonance in Medicine*, vol. 38, no. 1, pp. 117–128, 1997.

[20] K. A. Salem, H. Moriguchi, J. L. Duerk, and D. L. Wilson, "Optimization of noisy nonuniform sampling and image reconstruction for fast MRI using a human vision model," in *Medical Imaging 2001: Image Perception and Performance*, vol. 4324 of *Proceedings of SPIE*, pp. 82–90, San Diego, Calif, USA, February 2001.

[21] G. E. Sarty, R. Bennett, and R. W. Cox, "Direct reconstruction of non-cartesian *k*-space data using a nonuniform fast fourier transform," *Magnetic Resonance in Medicine*, vol. 45, no. 5, pp. 908–915, 2001.

[22] C. J. van den Branden Lambrecht and O. Verscheure, "Perceptual quality measure using a spatiotemporal model of the human visual system," in *Digital Video Compression: Algorithms and Technologies*, vol. 2668 of *Proceedings of SPIE*, pp. 450–461, San Jose, Calif, USA, January 1996.

[23] J. Lubin, "A visual discrimination model for imaging system design and evaluation," in *Vision Models for Target Detection and Recognition*, E. Peli, Ed., pp. 245–283, World Scientific, River Edge, NJ, USA, 1995.

[24] W. B. Jackson, M. R. Said, D. A. Jared, J. O. Larimer, J. L. Gille, and J. Lubin, "Evaluation of human vision models for predicting human observer performance," in *Medical Imaging 1997: Image Perception*, vol. 3036 of *Proceedings of SPIE*, pp. 64–73, Newport Beach, Calif, USA, February 1997.

[25] J. P. Johnson, J. Lubin, E. A. Krupinski, H. A. Peterson, H. Roehrig, and A. Baysinger, "Visual discrimination model for digital mammography," in *Medical Imaging 1999: Image Perception and Performance*, vol. 3663 of *Proceedings of SPIE*, pp. 253–263, San Diego, Calif, USA, February 1999.

[26] J. Oh, S. I. Woolley, T. N. Arvanitis, and J. N. Townend, "A multistage perceptual quality assessment for compressed digital angiogram images," *IEEE Transactions on Medical Imaging*, vol. 20, no. 12, pp. 1352–1361, 2001.

[27] S. Daly, "The visual differences predictor: an algorithm for the assessment of image fidelity," in *Digital Images and Human Vision*, A. B. Watson, Ed., pp. 179–206, MIT Press, Cambridge, Mass, USA, 1993.

[28] K. A. Salem, J. S. Lewin, A. J. Aschoff, J. L. Duerk, and D. L. Wilson, "Validation of a human vision model for image quality evaluation of fast interventional magnetic resonance imaging," *Journal of Electronic Imaging*, vol. 11, no. 2, pp. 224–235, 2002.

[29] K. A. Salem, J. L. Duerk, M. Wendt, and D. L. Wilson, "Evaluation of keyhole MR imaging with a human visual system response model," *Radiology*, vol. 209P, p. 246, 1998.

[30] C. A. Flask, K. A. Salem, H. Moriguchi, J. S. Lewin, D. L. Wilson, and J. L. Duerk, "Keyhole Dixon method for faster, perceptually equivalent fat suppression," *Journal of Magnetic Resonance Imaging*, vol. 18, no. 1, pp. 103–112, 2003.

[31] C. A. Flask, K. A. Salem, J. S. Lewin, and J. L. Duerk, "Keyhole Dixon method for faster fat suppression with perceptual equivalence," *Radiology*, vol. 225, p. 314, 2002.

[32] P. Xue, C. W. Thomas, G. C. Gilmore, and D. L. Wilson, "An adaptive reference/test paradigm: application to pulsed fluoroscopy perception," *Behavior Research Methods, Instruments, and Computers*, vol. 30, no. 2, pp. 332–348, 1998.

[33] Y. Jiang and D. L. Wilson, "Optimization of detector pixel size for stent visualization in x-ray fluoroscopy," *Medical Physics*, vol. 33, no. 3, pp. 668–678, 2006.

[34] R. Van de Walle, H. H. Barrett, K. J. Myers, et al., "Reconstruction of MR images from data acquired on a general nonregular grid by pseudoinverse calculation," *IEEE Transactions on Medical Imaging*, vol. 19, no. 12, pp. 1160–1167, 2000.

[35] K. F. King, T. K. F. Foo, and C. R. Crawford, "Optimized gradient waveforms for spiral scanning," *Magnetic Resonance in Medicine*, vol. 34, pp. 156–160, 1995.

[36] C. H. Hardy and H. E. Cline, "Broadband nuclear magnetic resonance pulses with two-dimensional spatial selectivity," *Journal of Applied Physics*, vol. 66, no. 4, pp. 1513–1516, 1989.

[37] A. Macovski, "Noise in MRI," *Magnetic Resonance in Medicine*, vol. 36, no. 3, pp. 494–497, 1996.

[38] D. Huo, D. Xu, Z.-P. Liang, and D. L. Wilson, "Application of perceptual difference model on regularization techniques of parallel MR imaging," *Magnetic Resonance Imaging*, vol. 24, no. 2, pp. 123–132, 2006.

[39] J. L. Mannos and D. J. Sakrison, "The effects of a visual fidelity criterion on the encoding of images," *IEEE Transactions on Information Theory*, vol. 20, no. 4, pp. 525–536, 1974.

[40] J. J. McCann, S. P. McKee, and T. H. Taylor, "Quantitative studies in retinex theory. A comparison between theoretical predictions and observer responses to the "color mondrian" experiments," *Vision Research*, vol. 16, no. 5, pp. 445–458, 1976.

[41] A. B. Watson, "The cortex transform: rapid computation of simulated neural images," *Computer Vision, Graphics, and Image Processing*, vol. 39, no. 3, pp. 311–327, 1987.

[42] R. A. Normann, B. S. Baxter, H. Ravindra, and P. J. Anderton, "Photoreceptor contributions to contrast sensitivity: applications in radiological diagnosis," *IEEE Transactions on Systems, Man and Cybernetics*, vol. 13, no. 5, pp. 944–953, 1983.

[43] ITU, "Recommendations ITU-R BT.500-11 Methodology for the subjective assessment of the quality of television pictures," 2002.

[44] J.-B. Martens and L. Meesters, "Image dissimilarity," *Signal Processing*, vol. 70, no. 3, pp. 155–176, 1998.

[45] A. E. Burgess, "Comparison of receiver operating characteristic and forced choice observer performance measurement methods," *Medical Physics*, vol. 22, no. 5, pp. 643–655, 1995.

[46] R. M. Manjeshwar and D. L. Wilson, "Effect of inherent location uncertainty on detection of stationary targets in noisy image sequences," *Journal of the Optical Society of America A*, vol. 18, no. 1, pp. 78–85, 2001.

[47] Y. Srinivas and D. L. Wilson, "Image quality evaluation of flat panel and image intensifier digital magnification in x-ray fluoroscopy," *Medical Physics*, vol. 29, no. 7, pp. 1611–1621, 2002.

[48] D. Green and J. A. Swets, *Signal Detection Theory and Psychophysics*, Krieger, New York, NY, USA, 1974.

[49] J. P. Johnson, K. J. Kirchberg, and C. H. Lorenz, "Perceptual evaluation of artifacts in cardiac magnetic resonance imaging due to partial parallel imaging," in *Medical Imaging 2005: Image Perception*, vol. 5749 of *Proceedings of SPIE*, pp. 549–556, San Diego, Calif, USA, February 2005.

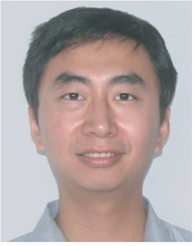

**Donglai Huo** received his B.S. and M.S. degrees in engineering physics from Tsinghua University, Beijing, China, in 1999 and 2001. Currently he is pursuing a Ph.D. degree in biomedical engineering at Case Western Reserve University. His research interests include image processing, image perception, and magnetic resonance imaging. In particular, he is working on the development and application of perceptual difference model (PDM) on spiral MR imaging and parallel MR imaging.

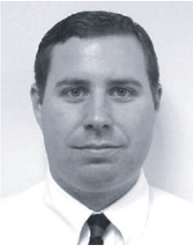

**Kyle A. Salem** received his B.S. and Ph.D. degrees in biomedical engineering from Case Western Reserve University. His thesis research focuses on the development of human visual system perceptual difference models for the evaluation of novel interventional MRI techniques. He worked in Siemens MRI Department after he received his Ph.D. degree in 2002, and then joined Cassling Diagnostic Imaging in 2004.

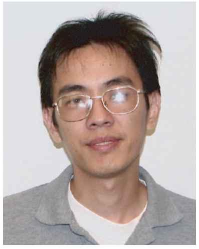

**Yuhao Jiang** received his Ph.D. degree in biomedical engineering at Case Western Reserve University in 2006. He received a M.S. degree in electrical engineering from Shanghai Jiaotong University, China, in 2000. His research interests are in the areas of digital image processing, X-ray physics, and visual perception studies for improving image quality in medical imaging. He has some publications in journals and conferences. He will start his job as an Assistant Professor at University of Central Oklahoma.

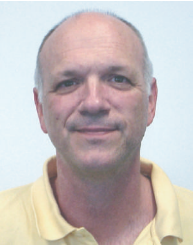

**David L. Wilson** he is a Professor of biomedical engineering and radiology, Case Western Reserve University, has interests in biomedical image processing, registration, and cellular and molecular imaging. He has a significant track record of federal research funding, over 65 refereed journal publications, and 7 patents. He has been active in the development of international conferences, including the IEEE, EMBS, and IEEE ISBI conferences. He has actively developed biomedical imaging at Case. He has led the BME faculty recruitment effort in imaging, and he has served as PI or has been an active leader on multiple research and equipment developmental awards to Case. Currently, he is the Case PI on the Ohio State University, Wright Center of Innovation award. The Biomedical Structural, Functional and Molecular Imaging Enterprise, an investment of more than 17M by the state of Ohio, which includes local industries such as Philips Medical Systems. Finally, he serves as Associate Chair in the Department of Biomedical Engineering.

